# Ropivacaine: A review of its pharmacology and clinical use

**DOI:** 10.4103/0019-5049.79875

**Published:** 2011

**Authors:** Gaurav Kuthiala, Geeta Chaudhary

**Affiliations:** Department of Anesthesia and Critical Care, SPS Apollo Hospitals, Ludhiana, India; 1 Department of Clinical Pharmacology, SPS Apollo Hospitals, Ludhiana, India

**Keywords:** Anaesthesia, regional anaesthetic, ropivacaine

## Abstract

Ropivacaine is a long-acting amide local anaesthetic agent and first produced as a pure enantiomer. It produces effects similar to other local anaesthetics via reversible inhibition of sodium ion influx in nerve fibres. Ropivacaine is less lipophilic than bupivacaine and is less likely to penetrate large myelinated motor fibres, resulting in a relatively reduced motor blockade. Thus, ropivacaine has a greater degree of motor sensory differentiation, which could be useful when motor blockade is undesirable. The reduced lipophilicity is also associated with decreased potential for central nervous system toxicity and cardiotoxicity. The drug displays linear and dose proportional pharmacokinetics (up to 80 mg administered intravenously). It is metabolised extensively in the liver and excreted in urine. The present article details the clinical applications of ropivacaine and its current place as a local anaesthetic in the group.

## INTRODUCTION

One of the most important properties of a long-acting local anaesthetic is to reversibly inhibit the nerve impulses, thus causing a prolonged sensory or motor blockade appropriate for anaesthesia in different types of surgeries.[1] The acute pain relief obtained at lower doses in postoperative and labour patients due to sensory blockade is sometimes marred by accompanying motor blockade, which serves no purpose and is quite undesirable.

Bupivacaine is a well-established long-acting regional anaesthetic, which like all amide anaesthetics has been associated with cardiotoxicity when used in high concentration or when accidentally administered intravascularly. Ropivacaine is a long-acting regional anaesthetic that is structurally related to Bupivacaine. It is a pure S(-)enantiomer, unlike Bupivacaine, which is a racemate, developed for the purpose of reducing potential toxicity and improving relative sensory and motor block profiles.[1]

## STEREOSPECIFICITY AND STRUCTURE

Enantiomers exist in two different spatial configurations, like right- and left-handed gloves, and are present in equal amounts in a racemic solution. They are optically active and can be differentiated by their effects on the rotation of the plane of a polarised light into dextrorotatory [clockwise rotation (R+)] or levorotatory [counterclockwise rotation (S-)] stereoisomers. The physicochemical properties of the two enantiomeric molecules are identical, but the two enantiomers can have substantially different behaviours in their affinity for either the site of action or the sites involved in the generation of side effects. R(+) and S(-) enantiomers of local anaesthetics have been demonstrated to have different affinity for different ion channels of sodium, potassium, and calcium; this results in a significant reduction in central nervous system (CNS) and cardiac toxicity (cardiotoxicity) of the S(-)enantiomer as compared with the R(+)enantiomer.[2]

The technological advancements have made it possible to develop Ropivacaine as an optically pure S(-) enantiomeric from the parent chiral molecule propivacaine. It belongs to the group of local anaesthetics, the pipecoloxylidides and has a propyl group on the piperidine nitrogen atom compared to bupivacaine, which has a butyl group.[3]

## MECHANISM OF ACTION

Ropivacaine causes reversible inhibition of sodium ion influx, and thereby blocks impulse conduction in nerve fibres.[1] This action is potentiated by dose-dependent inhibition of potassium channels.[4] Ropivacaine is less lipophilic than bupivacaine and is less likely to penetrate large myelinated motor fibres; therefore, it has selective action on the pain-transmitting A β and C nerves rather than Aβ fibres, which are involved in motor function.

## PHARMACODYNAMICS

### CNS and cardiovascular effects

Ropivacaine is less lipophilic than bupivacaine and that, together with its stereoselective properties,[5] contributes to ropivacaine having a significantly higher threshold for cardiotoxicity and CNS toxicity than bupivacaine in animals[5,6] and healthy volunteers.[7]

The lower lipophilicity of ropivacaine versus bupivacaine correlated with the lesser cardiodepressant effects of both ropivacaine isomers than of the bupivacaine isomers in animal studies.[5]

The CNS effects occurred earlier than cardiotoxic symptoms during an intravenous (IV) infusion of local anaesthetic (10 mg/min of ropivacaine or bupivacaine) in human volunteers and the infusion was stopped at this point. Significant changes in cardiac function involving the contractility, conduction time and QRS width occurred and the increase in a QRS width was found to be significantly smaller with ropivacaine than with bupivacaine.[8,9]

### Other effects

Ropivacaine has been shown to inhibit platelet aggregation in plasma at concentrations of 3.75 and 1.88 mg/mL (0.375% and 0.188%), which correspond to those that could occur in the epidural space during infusion.[10] Like other anaesthetics, ropivacaine has antibacterial activity *in vitro*, inhibiting the growth of *Staphylococcus aureus*,[11,12] *Escherichia coli*,[11] and *Pseudomonas aeruginosa*.[12]

## PHARMACOKINETICS

### Absorption and distribution

The plasma concentration of ropivacaine depends on the total dose administered and the route of administration, as well as the haemodynamic and circulatory condition of the patient and vascularity of the administration site.[13]

When ropivacaine was administered intravenously in subjects, its pharmacokinetics were linear and dose proportional up to 80 mg.[13] The absorption of ropivacaine 150 mg from the epidural space is complete and biphasic. The mean half-life of the initial phase is approximately 14 minutes, followed by a slower phase with a mean absorption t_1/2_ of approximately 4.2 hours.

Ropivacaine is bound to plasma proteins to an extent of 94%, mainly to α1-acid glycoprotein. The total plasma concentration increase during continuous epidural infusion of ropivacaine[13,14] is caused by an increase in the degree of protein binding and subsequent decrease in clearance of ropivacaine.[14]

Ropivacaine rapidly crosses the placenta during epidural administration for caesarean section, resulting in near complete equilibrium of the free fraction of ropivacaine in the maternal and foetal circulation.[15] However, the total plasma concentration of ropivacaine was lower in the foetal circulation than in the maternal circulation, reflecting the binding of ropivacaine to α1-acid glycoprotein, which is more concentrated in maternal than in foetal plasma.

### Metabolism and excretion

Ropivacaine is metabolised extensively in the liver, predominantly by aromatic hydroxylation to 3’-hydroxy-ropivacaine by cytochrome P450 (CYP) 1A2 and N-dealkylation to 2’,6’-pipecoloxylidide by CYP3A4.[16,17] The kidney is the main excretory organ for ropivacaine, accounting for 86% of the excretion of the drug in urine after a single intravenous dose administration. It has a mean ± SD terminal half-life of 1.8 ± 0.7 h and 4.2 ± 1.0 h after intravenous and epidural administration, respectively.

### Relative potency

A strict correlation exists between the lipid solubility of the local anaesthetic and its potency and toxicity. According to minimum local anaesthetic concentration (MLAC) studies, which are based on effective analgesia in 50% of patients) ropivacaine has similar potency to bupivacaine at higher doses (eg, doses required for peripheral nerve blocks for surgical anaesthesia), ropivacaine is less potent than bupivacaine and levobupivacaine at lower doses, such as those used for epidural or intrathecal analgesia. Providing anaesthesia or analgesia for the majority of patients is more clinically relevant than the MLAC and, at higher doses used in clinical practice, this potency difference is not always evident.

### Tolerability

Reactions to ropivacaine are characteristic of those associated with other amide-type local anaesthetics.

#### a. In adults

Ropivacaine is generally well tolerated regardless of the route of administration.[13] In a pooled analysis of data from controlled clinical trials adverse events that occurred in ≥5% of patients who received ropivacaine 0.125-1% via various routes of administration for surgery, labour, Caesarean section, postoperative pain management, peripheral nerve block or local infiltration (n=1,661) were hypotension (32%), nausea (17%), vomiting (7%), bradycardia (6%), and headache (5%).[13] These events are a consequence of nerve block and occurred with similar incidence in patients (n=1433) who received bupivacaine 0.25–0.75% for same indications (29%, 14%, 6%, 5%, and 5%, respectively).[13]

Epidural administration of ropivacaine for surgery generally produced dose-dependent adverse events similar to those observed with equal doses of bupivacaine.[13]

The incidence of ropivacaine-induced cardiovascular symptoms may be age-related;[18] patients aged ≥61 years who received epidural ropivacaine 1% had a significantly higher incidence of bradycardia ([58%] vs [15%] patients aged 41-60 years; P=0.005) and hypotension ([74%] vs [20%] patients aged 18-40 years; P=0.002).[18] The cardiovascular events are also related to toxicity due to sudden IV injection or massive absorption from peripheral nerve blocks.

#### b. In children

Ropivacaine was generally well tolerated in paediatric patients aged from 1 month to 15 years regardless of the route of administration.[19] The overall incidence of adverse events associated with ropivacaine appeared to be low,[19] with nausea and/or vomiting occurring most frequently.[19]

#### c. In exposed foetuses and neonates

Ropivacaine was generally well tolerated in the foetus or neonate following the use of regional anaesthesia in women undergoing Caesarean section or during labour.[13] The most common foetal or neonatal adverse events with ropivacaine were foetal bradycardia (12%), neonatal jaundice (8%), and unspecified neonatal complications (7%). These events occurred with similar frequency with bupivacaine (12%, 8%, and 7%, respectively).[13]

According to a meta-analysis of six double-blind trials, ropivacaine did not influence the neonatal neurological and adaptive capacity (NAC) score at 2 hours after delivery and, at 24 hours after delivery, total NAC scores were significantly higher in neonates whose mothers had received ropivacaine rather than bupivacaine (*P*<0.05).[20]

## CARDIOTOXICITY AND CNS TOXICITY IN COMPARISON TO BUPIVACAINE

The incidence of cardiotoxicity and central nervous system (CNS) toxicity as a result of inadvertent intravascular injection of ropivacaine appears to be low.[21] According to a pooled analysis of data from ≈3000 patients in 60 clinical studies, the incidence of probable accidental IV injection of ropivacaine was ≈0.2% (six patients) and only one patient experienced convulsions; no patient showed symptoms of cardiotoxicity.[21]

The convulsive local anaesthetic doses of bupivacaine and ropivacaine were studied in different animal models; bupivacaine has a 1.5- to 2.5-fold lower convulsive threshold when compared to ropivacaine. On the basis of animal and volunteer studies, it can be concluded that ropivacaine seems to be less neurotoxic and cardiotoxic than bupivacaine.

## DRUG INTERACTIONS

Ropivacaine should be used with caution in patients receiving other local anaesthetics or agents structurally related to amide-type local anaesthetics, since the toxic effects of these drugs are additive.

Cytochrome P4501A2 metabolises ropivacaine to 3-hydroxy ropivacaine, the major metabolite. Thus, strong inhibitors of cytochrome P4501A2, such as fluvoxamine, given concomitantly during administration of ropivacaine, can interact with ropivacaine and thus lead to increased ropivacaine plasma levels. Caution should be exercised when co-administering CYP1A2 inhibitors. Possible interactions with drugs known to be metabolised by CYP1A2 via competitive inhibition such as theophylline and imipramine may also occur.[22]

## DOSAGE

The dosage recommendations for ropivacaine in various indications and procedures are summarised in Table 1.

**Table 1:** Dosage recommendations for ropivacaine in adults and children

Indication in adults	Concentration (%)	Volume	Dose
In adults			
Surgical anaesthesia			
Lumbar epidural	0.75	15-20 mL	113-150 mg
(Caesarean section)			
Lumbar epidural	0.75	15-25 mL	113-188 mg
(Other surgery)	1	15-20 mL	150-200 mg
Thoracic (Single block for postoperative pain relief)	0.75	5-15 mL	38-113 mg
Intrathecal administration	0.5	3-4 mL	15-20 mg
Peripheral nerve block*	0.75	10-40 mL	75-300 mg
Field block†	0.75	1-30 mL	7.5-225 mg
Postoperative pain			
Lumbar epidural (Continuous infusion)	0.2	6-10 mL/h	12-20 mg/h
Thoracic epidural (Continuous infusion)	0.2	6-14 mL/h	12-28 mg/h
Peripheral nerve block (Continuous infusion)	0.2	5-10 mL/h	10-20 mg/h
Field block†	0.2	1-100 mL	2-200 mg
Intra-articular injection	0.75	20 mL	150 mg
Labour pain (Lumbar epidural)			
Bolus	0.2	10-20 mL	20-40 mg
Intermittent top-ups	0.2	10-15 mL‡	20-30 mg
Continuous infusion	0.2	6-14 mL/h	12-28 mg/h
In children			
Caudal epidural block (Below T12)§	0.2	1 mL/kg	2 mg/kg
Peripheral nerve block (Eg, ilioinguinal block)	0.5	0.6 mL/kg	3 mg/kg

* Major nerve block brachial plexus or sciatic nerve block

† Minor nerve block or infiltration

‡ Minimum interval 30 minutes

§ For bodyweight up to 25 kg.

## CLINICAL APPLICATIONS

Numerous clinical trials have evaluated the efficacy of ropivacaine for surgical anaesthesia, for labour pain and postoperative pain in adults and children. The drug has been compared primarily with bupivacaine or levobupivacaine. In the recent years, the use of ropivacaine in the management of chronic pain has also been evaluated in various modalities.

### Surgical anaesthesia

Clinical trials indicate that ropivacaine is an effective regional anaesthetic when administered via several routes.

### Epidural administration

Epidural ropivacaine, administered primarily in the lumbar region, has an effect of anaesthetic for a number of surgical procedures. A majority of studies on epidural ropivacaine are in Caesarean section and although the drug has been investigated as an anaesthetic agent for other abdominal or gynaecological procedures, orthopaedic and vascular surgery, the major use of epidural ropivacaine in the latter procedures is for postoperative pain relief.

#### a. Caesarean section

Clinical trials of epidural anaesthesia for elective Caesarean section indicate that ropivacaine (0.75% or 0.5%)[23] provides a clinically similar onset of sensory and motor block to that of bupivacaine 0.5%.[23] The median duration of analgesia within dermatomes relevant for surgery (T6–S3) was 1.7–4.2 hours for ropivacaine and 1.8–4.4 hours for bupivacaine, but the median duration of complete motor block was significantly longer with bupivacaine than with ropivacaine (2.5 vs 0.9 hours, *P*<0.05).[23]

#### b. Hip or lower limb surgery

In patients undergoing lumbar epidural anaesthesia for lower limb surgery, ropivacaine provided a similar anaesthetic profile (with regard to onset of analgesia or anaesthesia and onset of motor block) to those of levobupivacaine[24] or bupivacaine.[25] A 20-ml dose of ropivacaine 0.5% or bupivacaine 0.5% also resulted in a median duration of T10 sensory block of 3.5 versus 3.4 hours, and 15% versus 18% of patients with complete motor block.[25]

## INTRATHECAL ADMINISTRATION

Single doses of 2-4 ml of 0.5%-2% solutions of ropivacaine have been shown to be less potent than bupivacaine when administered intrathecally and is generally administered at a higher dose than bupivacaine. Hyperbaric solutions of ropivacaine have been compared to isobaric solution of the drug for various procedures and generally resulted in a faster onset and recovery from the blocks. Although hyperbaric solutions of ropivacaine appear to provide a more predictable block, the spread and duration of the block varies markedly. Hyperbaric ropivacaine solutions are not commercially available. Comparative trials in patients undergoing elective Caesarean section receiving ropivacaine 12 mg, levobupivacaine 8 mg, or bupivacaine 8 mg, all with sufentanil 2.5 μg showed similar times to onset of analgesia, but significantly shorter time to recovery from sensory and motor block was observed with ropivacaine and levobupivacaine as compared to bupivacaine.[26] The co-administration of opioids reduces the total dose of local anaesthetic required for anaesthesia and significantly prolongs the duration of complete and effective analgesia without prolonging the duration of motor block.[27]

In a trial involving lower limb surgeries the duration of sensory block with ropivacaine 15 mg was found to be similar with bupivacaine 10 mg, and the motor block was significantly shorter, it was also suggested that on a milligram for milligram basis, the potency of ropivacaine relative to bupivacaine is two-thirds with regard to sensory block and half with regard to motor block.[28]

## PERIPHERAL NERVE BLOCKS

Peripheral nerve block is employed for anaesthesia for orthopaedic surgery, and the onset and spread of local anaesthetic is influenced by the site of injection.[29] The long-acting sensory and motor block provided by ropivacaine is 0.5% or 0.75% for axillary, interscalene and subclavian perivascular brachial plexus block for hand or arm surgery compared favourably with bupivacaine 0.5%[29–33] or levobupivacaine 0.5% (30-45 ml bolus dose) with a similar quality of regional anaesthesia. In lower limb surgeries where sciatic or combined femoral and sciatic block was given for knee, ankle, or foot procedures, ropivacaine 0.75% (25 ml) had a significantly faster onset of sensory and motor block than 25 ml bupivacaine 0.5%. Although ropivacaine had a significantly shorter duration of sensory block, the duration of motor block remained similar with both agents.[34]

## MANAGEMENT OF POSTOPERATIVE PAIN

Lower doses of local anaesthetics are generally required for postoperative pain relief than for anaesthesia.

### Epidural administration

Ropivacaine is administered epidurally (via lumbar or thoracic route) for postoperative pain following abdominal (upper or lower), gynaecological, orthopaedic and other surgeries.

#### a. Following abdominal surgery

The efficacy of epidural ropivacaine has been compared with intravenous morphine, epidural bupivacaine, and ropivacaine in combination with fentanyl.

Ropivacaine, with or without morphine, was more effective at relieving postoperative pain than intravenous morphine alone.[35]

#### b. Following orthopaedic surgery

Patients who had undergone hip arthroplasty had significantly more effective pain relief with epidural ropivacaine than with intravenous morphine (primary endpoint) and supplementary analgesia was administered to numerically more patients in the morphine group than in the ropivacaine group.[36]

In patients who had undergone knee arthroplasty,[37] pain relief with epidural ropivacaine 0.2% was not as effective as bupivacaine 0.2%, and although there were no significant differences between groups for the number of patients recording visual analog scale (VAS) scores >30 mm at rest, more patients in the ropivacaine group than in the bupivacaine group reported VAS scores >30 mm for pain on movement during 8-24 hours post surgery. However, in another study comparing ropivacaine with bupivacaine in patients who had undergone hip arthroplasty,[38] the significantly lower incidence of motor block in ropivacaine recipients was accompanied by similarly effective pain relief among treatment groups and greater patient satisfaction.[38,39]

### Nerve blocks

#### a. Following upper limb surgery

There was similar pain relief with ropivacaine and bupivacaine,[40] although hand strength returned more quickly and there was less paraesthesia of the fingers in patients receiving ropivacaine than in those receiving bupivacaine.[40] At 24 hours after the block, hand strength (primary endpoint) reduced by 48% in ropivacaine recipients and 66% in bupivacaine recipients (*P*<0.05). Further, 6 hours after discontinuation of the infusion, hand strength was fully restored in the ropivacaine group but still decreased by 25% in the bupivacaine group (*P*<0.05).[40]

#### b. Following lower limb surgery

Patients who received combined femoral and sciatic nerve block[41] with ropivacaine to facilitate foot/ankle[41] surgery had similar or better postoperative pain relief and a longer duration of analgesia than recipients of mepivacaine.

## MANAGEMENT OF LABOUR PAIN

Epidurally administered ropivacaine is effective in providing relief from labour pain. It is recommended to administer 10-20 ml bolus of ropivacaine 0.2% with intermittent 20-30 mg top up injections or a continuous epidural infusion of ropivacaine 0.2% (6-10 ml/hr) for labour analgesia. The analgesic efficacy of ropivacaine is similar to or slightly less than bupivacaine. The difference between the incidences of operative deliveries when ropivacaine was compared with bupivacaine was also not found significant.

The addition of narcotics like fentanyl 2 *μ*g/ml to ropivacaine 0.1% solution administered at 10 ml/hr significantly reduces local anaesthetic concentration, as the quality of analgesia is similar to ropivacaine 0.2%-only solution or ropivacaine 0.2% plus fentanyl 2 μg/ml infused at a slower rate of 8 ml/hr.[42] Addition of adjuvants like clonidine also significantly increases the duration of action of ropivacaine.

Intrathecally administered ropivacaine as a part of combined spinal epidural technique produces rapid and effective labour pain relief with less incidence of motor block.[43]

## ROPIVACAINE AND CHRONIC PAIN MANAGEMENT

Lierz *et al*. compared the analgesic, motor block, and haemodynamic effects of single-shot epidural injections of ropivacaine 0.2% 10 mL with bupivacaine 0.125% in outpatients suffering from chronic low back pain in a randomised study involving 36 patients. Bupivacaine 0.125% and ropivacaine 0.2% showed no significant differences in analgesia or in motor blockade or haemodynamic changes. Both appear suitable for epidural administration to outpatients with chronic low back pain attending for epidural analgesia associated with physiotherapy.[44] In another study, the prophylactic effectiveness of ropivacaine injections in migraine was studied during a 12-week period. A total of 52 patients participated in the study. Trigger points were explored by palpitation and injected weekly with 10 mg ropivacaine. In nine patients (17.3%), the frequency of attacks was reduced ≥50%, and in 19 cases (36.5%), the reduction was between 11% and 49%. A total of 31 patients (59.6%) reported to be much or very much improved after completing the injection period. Trigger points inactivation can be an effective palliative measure in the prophylactic management of severe refractory migraine.[45]

## CONCLUSION

Ropivacaine is a well tolerated regional anaesthetic effective for surgical anaesthesia as well as the relief of postoperative and labour pain. The efficacy of ropivacaine is similar to that of bupivacaine and levobupivacaine for peripheral nerve blocks and, although it may be slightly less potent than bupivacaine when administered epidurally or intrathecally, equi-effective doses have been established. Clinically adequate doses of ropivacaine appear to be associated with a lower incidence or grade of motor block than bupivacaine. Thus, ropivacaine, with its efficacy, lower propensity for motor block, and reduced potential for CNS toxicity and cardiotoxicity, appears to be an important option for regional anaesthesia and management of postoperative and labour pain.
